# Real-World Effectiveness of IHAT^TM^ in Women with Iron Deficiency Across Pregnancy, Postpartum and Abnormal Uterine Bleeding

**DOI:** 10.3390/nu18152446

**Published:** 2026-07-27

**Authors:** Gabriele Saccone, Mariarosaria Motta, Francesco De Seta, Salvatore Mola, Sara Iannantuoni, Nicoletta Di Simone

**Affiliations:** 1 Department of Neuroscience, Reproductive Science and Dentistry, School of Medicine, University of Naples Federico II, 80131 Naples, Italy; 2 Department of Obstetrics and Gynecology, IRCCS San Raffaele Scientific Institute, University Vita and Salute, 20132 Milan, Italy; fradeseta@gmail.com; 3 Department of Biomedical Sciences, Humanitas University, 20072 Milan, Italy

**Keywords:** iron deficiency, iron deficiency anemia, pregnancy, postpartum period, abnormal uterine bleeding, oral iron supplementation, Iron Hydroxide Adipate Tartrate, IHAT^TM^, real-world study, treatment adherence

## Abstract

**Objective**: To evaluate the real-world effectiveness and tolerability of Iron Hydroxide Adipate Tartrate (IHAT^TM^) in women with iron deficiency across three common obstetric and gynecologic clinical settings: pregnancy, the postpartum period, and abnormal uterine bleeding. **Methods**: This prospective multicenter observational real-world study included women with iron deficiency managed in routine clinical practice across Italy. Participating gynecologists collected data using standardized case report forms. Patients received IHAT^TM^ according to routine clinical practice and were followed for approximately three months. Baseline demographic and hematologic parameters were recorded, including hemoglobin and ferritin levels. The primary outcome was the change in hemoglobin between baseline and follow-up. Secondary outcomes included changes in ferritin levels, treatment adherence, and tolerability. **Results**: A total of 517 women was included in the analysis. The study population included pregnant women (*N* = 287, 55.5%), women with abnormal uterine bleeding (*N* = 161, 31.1%), and women in the postpartum period (*N* = 69, 13.3%). Improvements in hemoglobin were observed across all clinical settings, with hemoglobin increasing from 10.35 g/dL at baseline to 11.63 g/dL at three months, corresponding to a mean increase of 1.28 g/dL. Mean ferritin levels increased from 22.50 ng/mL to 37.74 ng/mL in three months. Treatment adherence was high or moderate in 93.4% of patients. Gastrointestinal adverse events were infrequent, with constipation (9.7%) and nausea (4.8%) among the most reported. **Conclusions**: In this prospective real-world multicenter study, treatment with IHAT^TM^ was associated with meaningful improvements in hemoglobin and iron stores in women with iron deficiency across pregnancy, postpartum, and abnormal uterine bleeding. The treatment showed good adherence and a favorable tolerability profile in routine clinical practice.

## 1. Introduction

Iron deficiency represents not only a hematological condition but also a broader systemic disorder that can significantly impact quality of life, cognitive function, and physical performance in women of reproductive age [1,2,3]. Even in the absence of overt anemia, iron depletion has been associated with fatigue, reduced exercise tolerance, impaired immune response, and decreased work productivity. These clinical manifestations are particularly relevant in reproductive-aged women, who often experience cumulative iron loss over time due to physiological and pathological processes.

From a public health perspective, iron deficiency remains one of the most prevalent nutritional deficiencies worldwide, with prevalence estimates ranging from 20% to over 40% in women of reproductive age depending on geographic and socioeconomic factors [4,5]. Despite the availability of multiple therapeutic strategies, optimal management of iron deficiency continues to represent a challenge in routine clinical practice, especially in settings where long-term adherence to therapy is required [6].

Several physiological and pathological conditions can contribute to reduced iron stores in this population. Increased iron requirements during pregnancy, blood loss related to delivery in the postpartum period, and chronic menstrual bleeding in women with abnormal uterine bleeding are among the most common contributors to iron deficiency in these settings [7].

During pregnancy, maternal iron demand increases progressively as a consequence of expanded red cell mass and the requirements of the developing fetus and placenta. When iron intake or absorption is insufficient to meet these demands, iron deficiency and anemia may develop. Postpartum women may also experience depletion of iron stores, particularly following peripartum blood loss. In the gynecologic population, abnormal uterine bleeding represents a major cause of chronic iron loss and is frequently associated with iron deficiency and iron deficiency anemia.

Oral iron supplementation is generally recommended as first-line therapy for iron deficiency in many clinical contexts, although variability in absorption and response to treatment has been widely documented [8,9,10]. Furthermore, conventional oral iron preparations are often associated with gastrointestinal adverse effects that can limit treatment adherence [11].

In addition to gastrointestinal intolerance, conventional oral iron formulations may be limited by suboptimal absorption in specific clinical conditions characterized by inflammation or altered intestinal function. Elevated hepcidin levels, commonly observed during pregnancy and in inflammatory states, may further reduce iron bioavailability by inhibiting intestinal iron transport mechanisms. Consequently, even when oral iron is prescribed appropriately, the expected hematologic response may not always be achieved, particularly in patients with underlying gastrointestinal disorders or chronic inflammatory conditions.

In routine clinical practice, intolerance to therapy and poor compliance may reduce the overall effectiveness of oral iron supplementation.

Iron Hydroxide Adipate Tartrate (IHAT^TM^) is a novel food and a next-generation oral iron source designed to deliver iron in a nanoparticulate form that closely mimics the mineral core of ferritin [12], representing a clear advancement over conventional supplements.

Following EFSA’s comprehensive safety and bioavailability assessment [13], IHAT^TM^ has been proposed as an effective strategy to enhance iron delivery while overcoming key limitations of traditional iron salts, particularly gastrointestinal intolerance.

**Figure 1:**
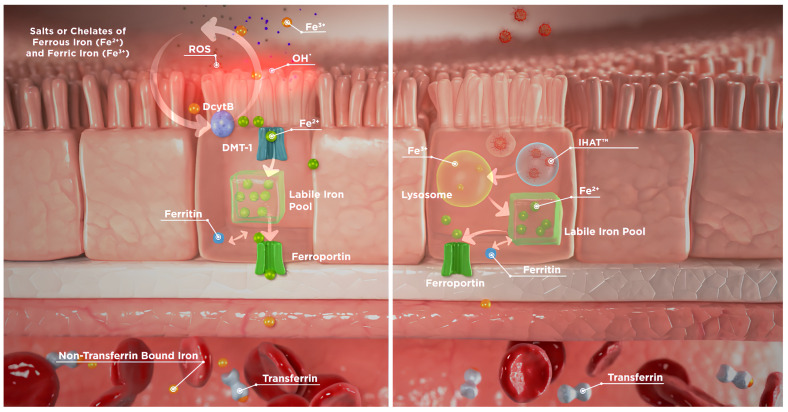
Representation of the absorption mechanism of IHAT™ compared to conventional iron forms.

IHAT™ features a novel absorption mechanism via endocytosis [14], as illustrated in Figure 1, enabling iron uptake without releasing free iron in the intestinal lumen and thereby avoiding redox reactions responsible for gastrointestinal damage [15,16]. This uptake pathway appears to be less influenced by inflammatory states and elevated hepcidin levels, which typically limit iron absorption [17,18]. In preclinical studies, IHAT™ promotes a favorable gut microbiota, preserving beneficial microorganisms (Lactobacillus) and inhibiting the growth of pathogenic bacteria (Bacteroides and Echerichia) [15], unlike ferrous sulfate, as the absence of free luminal iron contributes to a stable microbial ecosystem. Moreover, IHAT^TM^ provides a controlled and progressive iron availability, avoiding tissue accumulation in the intestinal MALT (Mucosa-Associated Lymphoid Tissue), liver, and spleen [13], and reducing the risk of non-physiological plasma iron peaks, further supporting its safety and tolerability [15]. Previous clinical studies have shown that IHAT™ is effective in improving iron status and is generally well tolerated in patients with iron deficiency, with randomized trials reporting efficacy comparable to ferrous sulfate together with an improved gastrointestinal tolerability profile. However, clinical evidence in obstetric and gynecologic populations, particularly during pregnancy and the postpartum period, remains limited, and real-world data in these settings are still scarce. Real-world studies may help clarify how this therapy performs outside controlled research settings and in heterogeneous patient populations [19].

### Objective

The objective of the present study was to evaluate the real-world effectiveness of IHAT^TM^ in women with iron deficiency across three common clinical settings in obstetric and gynecologic practice: pregnancy, the postpartum period, and abnormal uterine bleeding.

## 2. Methods

### 2.1. Methods Study Design and Setting

This was a prospective, multicenter, real-world observational study conducted in routine obstetric and gynecologic clinical practice across Italy. The study involved a network of participating obstetricians and gynecologists practicing in both hospital and private clinical settings across multiple Italian cities, all of whom contributed prospectively collected real-world clinical data using standardized case report forms.

The study aimed to evaluate the effectiveness of IHAT^TM^ in women with iron deficiency managed in three common clinical settings: pregnancy, the postpartum period, and gynecologic patients with abnormal uterine bleeding.

Because treatment decisions were made according to routine clinical practice and no experimental intervention was imposed by the study protocol, the investigation was conducted as a non-interventional observational study.

### 2.2. Study Population

Women with iron deficiency who were prescribed IHAT^TM^ in routine clinical practice were eligible for inclusion. Iron deficiency was diagnosed according to routine clinical practice based on laboratory parameters including serum ferritin, hemoglobin, serum iron, and transferrin saturation, interpreted by the treating physician.

Patients were enrolled consecutively by participating clinicians and categorized into three predefined clinical groups: pregnant women (1st, 2nd, and 3rd trimester), women in the postpartum period, and gynecologic patients with abnormal uterine bleeding. A total of 517 patients were included in the study database.

### 2.3. Data Collection

Clinical data were prospectively recorded using standardized case report forms completed by the treating physicians. Information collected at baseline included demographic characteristics, clinical setting, and laboratory parameters related to iron status. Baseline hematologic variables included hemoglobin (Hb), ferritin, serum iron, transferrin saturation (TSAT), red blood cell count (RBC), and mean corpuscular volume (MCV) when available. Treatment information included prescription of commercially available Iron Hydroxide Adipate Tartrate (IHAT™; Enteralia Bioscience S.r.l., Monza, Italy). IHAT^TM^ was prescribed according to routine clinical practice at a daily dose of 30, 60, or, in a limited number of cases, 90 mg of elemental iron. Dose selection was left to the treating physician and was based on the clinical severity of iron deficiency, baseline hematologic parameters, clinical setting (pregnancy, postpartum, or abnormal uterine bleeding), and overall clinical judgment. Patients were instructed to take IHAT™ once daily, in the morning on an empty stomach, according to the manufacturer’s recommendations. Patients were followed according to routine clinical practice, and follow-up data were collected approximately three months after treatment initiation. According to routine clinical practice, concomitant treatments could include folic acid, prenatal multivitamins, progesterone therapy, hormonal treatment for abnormal uterine bleeding, and other medications routinely prescribed according to the underlying clinical condition. No clinically relevant interactions with IHAT™ have been reported. Patients were followed according to routine clinical practice, and follow-up data were collected approximately three months after treatment initiation.

### 2.4. Outcomes

The primary objective of the study was to assess the real-world effectiveness of IHAT^TM^ by evaluating changes in hematologic parameters between baseline and follow-up. Secondary outcomes included evaluation of treatment adherence, physician-reported clinical response, and tolerability of therapy during the observation period.

### 2.5. Statistical Analysis

Continuous variables were summarized using the mean and standard deviation or the median and interquartile range, depending on data distribution. Categorical variables were reported as frequencies and percentages. Changes in hematologic parameters between baseline and follow-up were evaluated using a paired Student’s *t*-test. Statistical analyses were performed for the overall study population and within each treatment group according to clinical setting and IHAT™ dosing regimen. Results are reported as *t* statistics and corresponding two-sided *p* values. A two-sided *p* value < 0.05 was considered statistically significant. Subgroup analyses were performed according to the three predefined clinical settings (pregnancy, postpartum, and abnormal uterine bleeding) and to the dosing regimen (30 mg daily or 60 mg daily). All analyses were conducted using the available study database, including 517 patients.

Given the real-world observational design of the study, no predefined sample size calculation was performed. The study aimed to include all eligible patients treated with IHAT^TM^ within the participating centers during the study period in order to reflect routine clinical practice as closely as possible.

Missing data were managed according to available-case analysis, and no imputation methods were applied. The observational nature of the study implies that variations in clinical management, laboratory assessment timing, and follow-up procedures may have occurred across participating centers, reflecting real-life heterogeneity in clinical practice.

## 3. Results

### 3.1. Study Population

A total of *N* = 517 women were included in the study. Patients were distributed across the three predefined clinical settings as follows: pregnancy (*N* = 287, 55.5%), abnormal uterine bleeding (*N* = 161, 31.1%), and postpartum (*N* = 69, 13.3%).

According to the provided dose regimen (expressed as elemental iron), the patients were distributed as follows: 30 mg daily (*N* = 339, 65.6%), 60 mg daily (*N* = 172, 33.3%), and 90 mg daily (*N* = 6, 1.2%). Baseline demographic and clinical characteristics of the study population are reported in Table 1.

### 3.2. Hematologic Parameters

**Figure 2:**
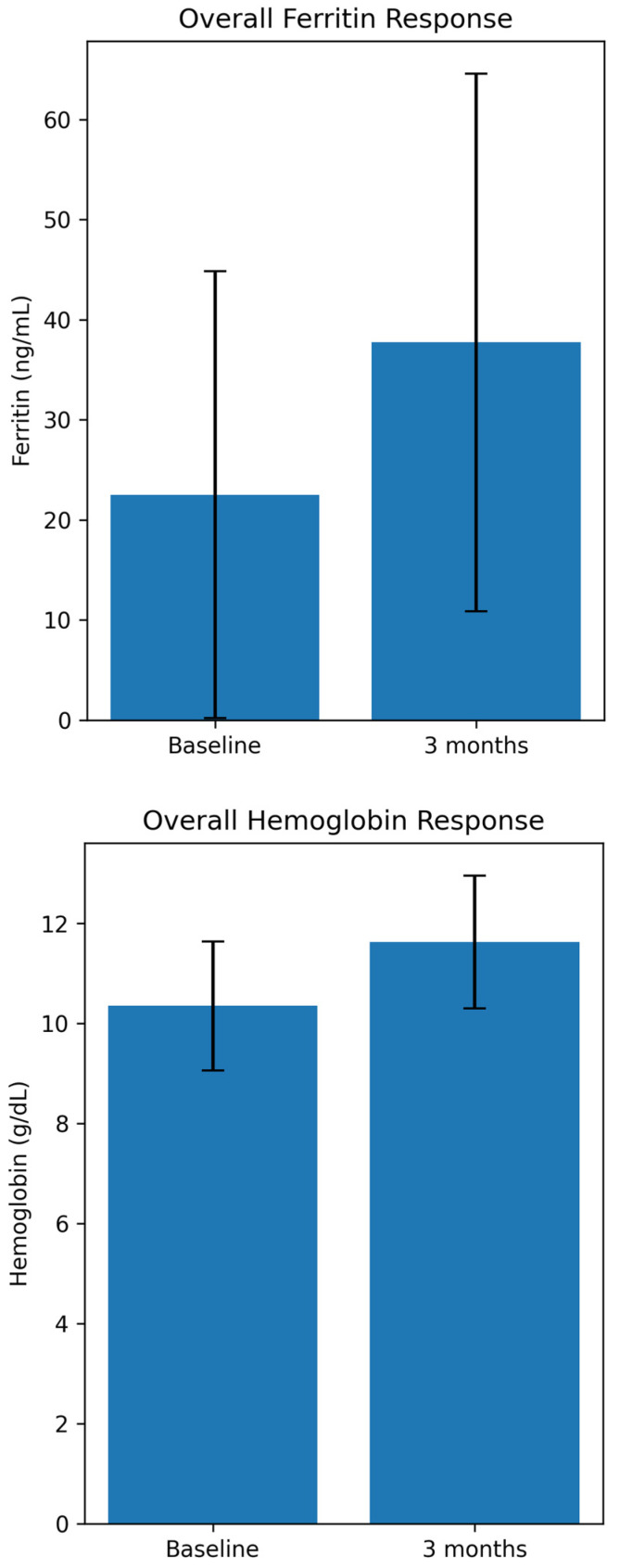
Changes in hematologic parameters from baseline to 3 months.

**Figure 3:**
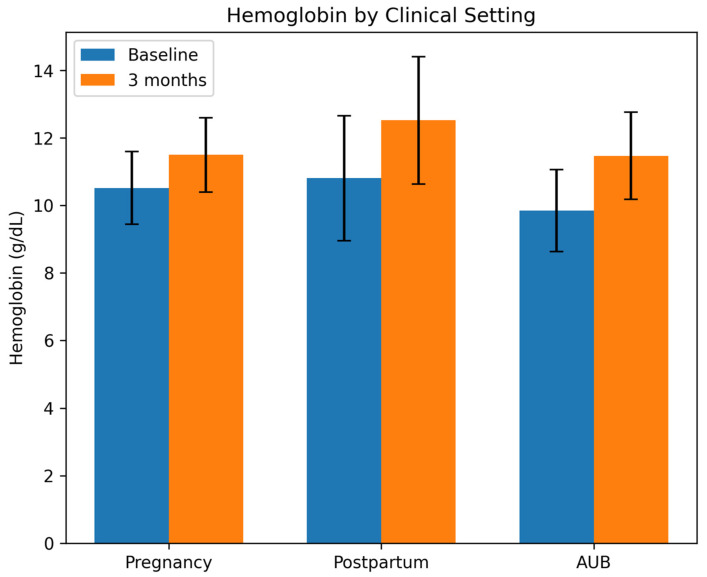
Hemoglobin response stratified by clinical setting.

**Figure 4:**
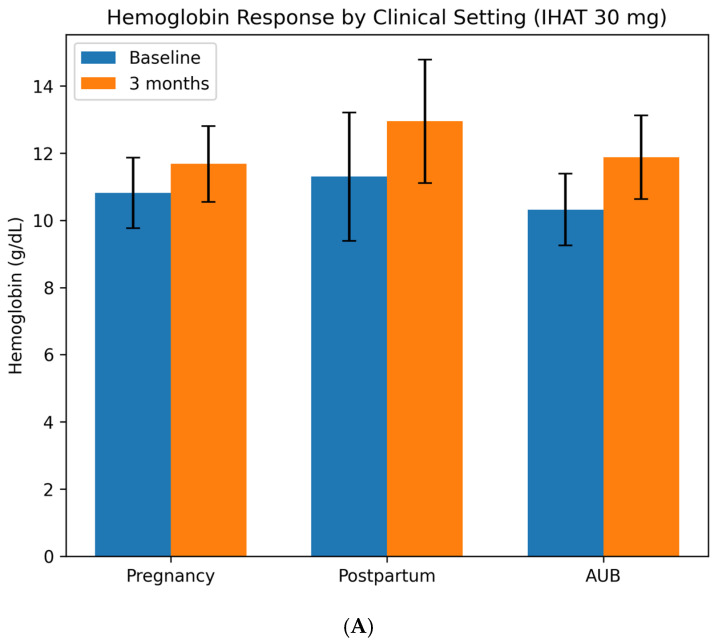

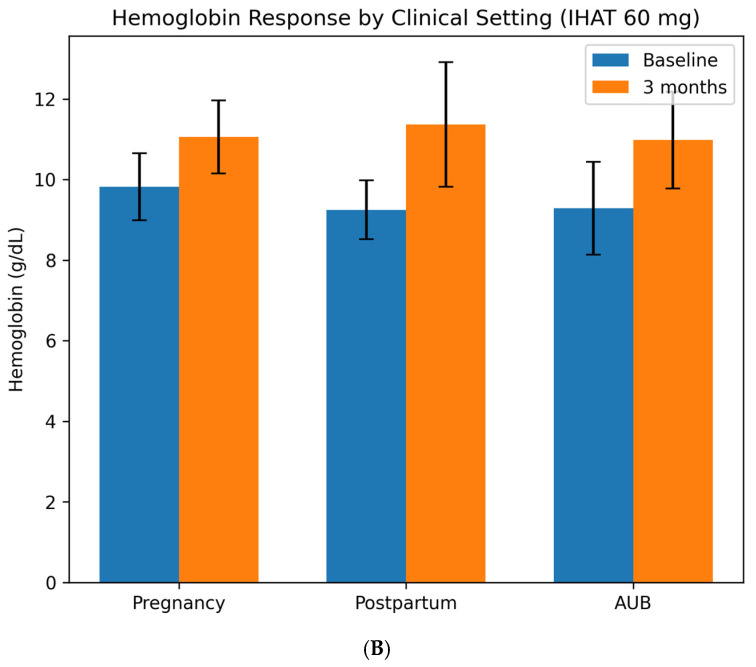
Change in hemoglobin parameters from baseline to 3 months by dosing regimen stratified by clinical setting. (**A**) IHATTM 30 mg supplementation; (**B**) IHAT^TM^ 60 mg supplementation.

At baseline, the mean Hb level was 10.35 g/dL (SD 1.29). At 3 months of IHAT^TM^ supplementation, mean hemoglobin was 11.63 g/dL (SD 1.33), a mean change in Hb of +1.28 g/dL (SD 1.13), which was statistically significant (paired Student’s *t*-test, *p* < 0.001). Iron stores also improved: mean serum ferritin levels increased from 22.50 ng/mL (SD 22.3) at baseline to 37.74 ng/mL (SD 26.85) at 3 months, with a mean serum ferritin increase of +15.24 ng/mL (SD 26.64) (*p* < 0.001). Increases in additional, related hematologic parameters, including serum iron, TSAT, RBC, and mean MCV, were also observed and are summarized in Table 2. Data stratified by clinical setting and dose regimen are presented in Table 2, Table 3, Table 4 and Table 5, Figure 2, Figure 3 and Figure 4. Within the overall *N* = 517 women, *N* = 15 subjects with gastrointestinal comorbidities such as inflammatory bowel disease (IBD), irritable bowel syndrome (IBS), celiac disease (CeD), and prior bariatric surgery were included. In this subgroup, where iron absorption with conventional oral therapies is impaired, an improvement in hematological parameters was also observed. At baseline, the mean Hb level was 10.14 g/dL (SD 1.15), and at 3 months after IHAT^TM^ supplementation, mean Hb increased to 11.26 g/dL (SD 0.99), with a mean increase of +1.12 g/dL (SD 1.02). Serum ferritin was 17.00 ng/mL (SD 13.70) at baseline and 24.45 ng/mL (SD 16.06) at 3 months, with a mean increase of +7.45 ng/mL (SD 17.50).

### 3.3. Symptom Resolution

As per physician-reported evaluation, clinical symptoms associated with ID such as fatigue and asthenia showed improvement with a substantial proportion of patients reporting resolution or significant improvement after IHAT^TM^ intake.

### 3.4. Treatment Adherence

Adherence to treatment was reported as high adherence (*N* = 350, 67.7%), moderate adherence (*N* = 133, 25.7%), and low adherence (*N* = 34, 6.6%).

Overall, the majority of patients demonstrated high or moderate adherence to therapy (93.4%).

### 3.5. Tolerability

IHAT^TM^ was generally well tolerated. Reported gastrointestinal adverse events were uncommon and included: 9.7% constipation (*N* = 50), 4.8% nausea (*N* = 25), 3.3% reflux (*N* = 17), 2.5% abdominal pain (*N* = 13), 1.4% diarrhea (*N* = 7), 0.4% vomiting (*N* = 2). No severe adverse events were reported in the study database.

### 3.6. Physician Evaluation

**Figure 5:**
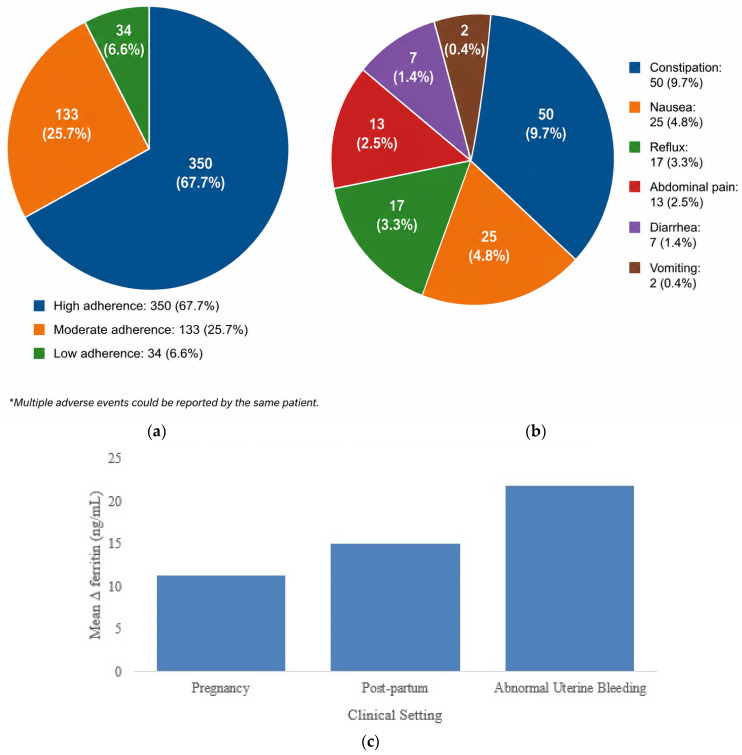
Treatment adherence and tolerability. (**a**) Treatment adherence (*N* = 517), (**b**) Tolerability (*N* = 114), (**c**) Change in ferritin at 3 months by clinical setting.

At the end of the observation period, physicians reported an overall improvement in hematologic parameters in the majority of patients receiving IHAT^TM^. Detailed physician assessments are summarized in Figure 5.

## 4. Discussion

### 4.1. Main Findings

In this prospective multicenter real-world study, including 517 women with iron deficiency managed in routine obstetric and gynecologic institutional and private office practice, treatment with IHAT^TM^ was associated with a clinically meaningful improvement in hematologic parameters after three months of IHAT^TM^ supplementation.

Overall, hemoglobin levels increased substantially between baseline and follow-up at 3 months of supplementation, with a mean increase of approximately 1.3 g/dL in the overall population. Improvement in iron stores was also observed, as reflected by the increase in ferritin concentrations. These findings were consistent across the three predefined clinical settings included in the study, i.e., pregnancy, postpartum, and abnormal uterine bleeding. The magnitude of hemoglobin improvement differed among clinical settings, with larger increases observed in women in the postpartum period and in those with abnormal uterine bleeding compared with pregnant women. In pregnant women, greater Hb and serum ferritin improvement were observed in those patients provided with a 60 mg iron equivalent daily dose. This pattern is biologically plausible, as iron requirements and baseline iron status may differ between these different clinical settings [20]. Treatment adherence was generally high, with more than two-thirds of patients reporting high adherence to therapy and more than 90% reporting at least moderate adherence. In addition, the treatment was well tolerated, with relatively low rates of gastrointestinal adverse events reported in routine clinical practice. Of interest, the Hb and serum ferritin increase observed in a small subgroup of patients with gastrointestinal inflammatory comorbidities, commonly regarded as non-responders to conventional oral iron supplementation and often requiring parenteral iron interventions. However, these findings should be considered exploratory because of the limited number of patients and the heterogeneity of the gastrointestinal conditions represented. Larger dedicated studies are warranted to further investigate the potential role of IHAT™ in this patient population. 

Taken together, these findings suggest that IHAT^TM^ represents an effective and highly tolerated oral treatment option for iron deficiency in several common obstetric and gynecologic clinical contexts and may represent a promising treatment option for patients with impaired oral iron absorption due to inflammatory bowel disease. However, dedicated comparative studies are needed to confirm this potential role.

Several limitations should be acknowledged: (i) the study was observational and did not include a control group receiving an alternative iron formulation. Therefore, causal inference regarding comparative effectiveness cannot be established; (ii) treatment decisions were made according to routine clinical practice and laboratory assessments were not performed within a strictly standardized research protocol; (iii) another limitation is the relatively short duration (3 months) of treatment, which may not fully capture long-term outcomes or recurrence of iron deficiency; (iv) some degree of heterogeneity in clinical management across participating physicians cannot be excluded, as always happens with many real-world observational studies; furthermore, laboratory analyses, including ferritin measurements, were performed locally according to routine clinical practice rather than in a centralized laboratory, consequently, some degree of inter-laboratory variability cannot be excluded; (v) spontaneous recovery of hemoglobin levels, particularly in postpartum women, may have contributed to the observed hematologic improvements. Therefore, the treatment effect cannot be fully distinguished from the natural course of recovery in this subgroup; (vi) a formal screening log was not maintained during patient recruitment. Therefore, the number of patients screened, excluded, and the reasons for exclusion could not be systematically documented, precluding the construction of a STROBE flow diagram; (vii) the available dataset did not include sufficient information to perform multivariable regression analyses adjusting for potential confounding factors, and, for this reason, residual confounding cannot be excluded, and the observed associations should be interpreted with appropriate caution.

### 4.2. Comparison with Existing Literature

The findings of the present study are consistent with previous evidence suggesting that IHAT may represent a promising oral iron formulation with a favorable tolerability profile, although no direct comparison with conventional oral iron preparations was performed in the present study [8,21,22]. While randomized controlled trials remain the gold standard for establishing efficacy, real-world studies provide complementary information by capturing treatment performance in broader and more heterogeneous populations.

In particular, adherence to oral iron therapy is a well-recognized determinant of treatment success. Previous studies have reported discontinuation rates of conventional oral iron therapy ranging from 20% to 50%, primarily due to gastrointestinal side effects [23,24]. In this context, the high adherence rates observed in the present study may represent a clinically relevant finding, suggesting that improved tolerability could translate into better overall treatment effectiveness. Because the present study did not include an active comparator, our findings should be interpreted as evidence of the real-world performance of IHAT™ rather than as proof of superiority over conventional oral iron formulations.

### 4.3. Implication

Iron deficiency is frequently encountered in both obstetric and gynecologic care and often requires pharmacologic treatment [25]. However, intolerance to conventional oral iron preparations may represent a significant barrier to effective therapy [26]. The results of this study suggest that IHAT^TM^ may represent a practical treatment option in routine clinical settings, demonstrating improvement in hemoglobin levels and iron stores together with a favorable tolerability profile. Indeed, the relatively high adherence observed in this cohort reflects acceptable tolerability in daily clinical practice. These findings may be particularly relevant in clinical scenarios where oral iron therapy is preferred, but gastrointestinal iron intolerance to other oral iron forms limits adherence. In such contexts, IHAT^TM^ may help improve treatment efficacy.

Further research is needed to better define the role of IHAT^TM^ in the management of iron deficiency across different patient populations. Future studies could include randomized comparative trials evaluating IHAT^TM^ against conventional oral iron formulations in specific obstetric or gynecologic populations. In addition, longer-term observational studies may help assess the durability of hematologic response and the potential impact on patient-reported outcomes such as fatigue and quality of life. Additional research may also explore optimal dosing strategies and treatment duration in different clinical settings, including pregnancy and postpartum care.

### 4.4. Clinical Relevance

From a clinical perspective, the management of iron deficiency in women of reproductive age requires a balance between efficacy, tolerability, and patient adherence. In pregnancy and postpartum settings, timely correction of iron deficiency is particularly important to prevent adverse maternal and neonatal outcomes. In obstetric populations, untreated iron deficiency has been associated with adverse outcomes including preterm delivery, low birth weight, and impaired maternal recovery in the postpartum period [27,28,29]. Similarly, in women with abnormal uterine bleeding, chronic iron loss may lead to persistent or recurrent anemia if not adequately addressed.

The results of this study suggest that IHAT^TM^ may represent a valuable therapeutic option in these contexts, particularly for patients who experience intolerance to conventional oral iron or who require prolonged treatment. The observed improvements in hematologic parameters, combined with a favorable tolerability profile, support its potential role in routine clinical practice.

## 5. Conclusions

In this prospective multicenter real-world study involving 517 women with iron deficiency, treatment with IHAT^TM^ was associated with a significant improvement in hemoglobin levels and iron stores after three months of therapy. As a next-generation oral iron and novel food, IHAT^TM^ demonstrated high adherence and a good tolerability profile in routine clinical practice. The favorable tolerability is related to its unique mechanism of action, which delivers iron in a protected form without releasing free luminal iron, which is responsible for damage to the intestinal mucosa and helps preserve gut microbiota balance. Collectively, these findings suggest that IHAT^TM^ represents an effective, well-tolerated, and innovative oral treatment option for iron deficiency in women during pregnancy, postpartum, and in the context of abnormal uterine bleeding. Future research should further explore the long-term clinical impact of IHAT^TM^, including its role in preventing recurrence of iron deficiency and its potential benefits in specific high-risk populations.

## Figures and Tables

**Table 1:** Characteristics of the study population.

	Value
**Total patients**	517
Clinical setting—Pregnancy	287 (55.5%)
Clinical setting—Postpartum	69 (13.3%)
Clinical setting—Abnormal uterine bleeding	161 (31.1%)
Age 18–25 years	70 (13.5%)
Age 26–35 years	209 (40.4%)
Age 36–45 years	156 (30.2%)
Age > 45 years	82 (15.9%)
No comorbidities	369 (71.4%)
Gastrointestinal disorders	41 (7.9%)
Autoimmune diseases	27 (5.2%)
Hematological disorders-coagulopathies	18 (3.5%)
Other conditions	28 (5.4%)
Concomitant therapies	228 (44.1%)
30 mg dose regimen	339 (65.6%)
60 mg dose regimen	172 (33.3%)
90 mg dose regimen	6 (1.2%)

**Table 2:** Changes in hematologic parameters from baseline to 3 months.

	Baseline Mean (SD)	3 Months Mean (SD)	Mean Change (SD)
**Hemoglobin (g/dL)**	10.35 (1.29)	11.63 (1.33)	1.28 (1.13)
**Ferritin (ng/mL)**	22.50 (22.32)	37.74 (26.85)	15.24 (26.64)
**Serum Iron (µg/dL)**	50.82 (27.81)	74.32 (31.15)	23.50 (31.70)
**TSAT (%)**	18.86 (13.29)	30.02 (13.60)	11.16 (14.00)
**RBC (10^12^/L)**	3.86 (0.68)	4.31 (0.63)	0.45 (0.52)
**MCV (fL)**	81.80 (9.62)	85.67 (6.40)	3.87 (8.40)

**Table 3:** Changes in hematologic parameters from baseline to 3 months by dosing regimen.

	IHAT^TM^ 30 mg (*N*. 339)			IHAT^TM^ 60 mg (*N*. 172)		
	Baseline Mean (SD)	3 Months Mean (SD)	Mean Change (SD)	Baseline Mean (SD)	3 Months Mean (SD)	Mean Change (SD)
**Hemoglobin (g/dL)**	10.76 (1.24)	11.92 (1.34)	1.16 (1.24)	9.57 (0.99)	11.06 (1.12)	1.49 (0.85)
**Ferritin (ng/mL)**	25.46 (25.53)	37.09 (28.26)	11.63 (29.03)	17.03 (13.55)	39.09 (24.56)	22.05 (20.35)

**Table 4:** Changes in hematologic parameters from baseline to 3 months by IHAT™ dosing regimen.

Group	*t*	*p*
Hb (IHAT^TM^ 30 mg)	17.22	<0.0001 *
Hb (IHAT^TM^ 60 mg)	22.99	<0.0001 *
Ferritin (IHAT^TM^ 30 mg)	7.38	<0.0001 *
Ferritin (IHAT^TM^ 60 mg)	14.21	<0.0001 *

Data were analyzed using a paired Student’s *t*-test comparing baseline and 3-month values within each dosing group. *t* = Student’s *t* statistic; *p* = two-sided *p* value. Hb, hemoglobin. * Denotes statistically significant *p*-values (*p* < 0.05).

**Table 5:** Paired Student’s *t*-test for changes in hemoglobin according to clinical setting and IHAT™ dosing regimen.

Group	*t*	*p*
Pregnancy (IHAT^TM^ 30 mg)	11.08	<0.0001 *
Pregnancy (IHAT^TM^ 60 mg)	16.14	<0.0001 *
Postpartum (IHAT^TM^ 30 mg)	8.45	<0.0001 *
Postpartum (IHAT^TM^ 60 mg)	6.20	<0.0001 *
AUB (IHAT^TM^ 30 mg)	11.52	<0.0001 *
AUB (IHAT^TM^ 60 mg)	17.80	<0.0001 *

Statistical comparisons were performed using a paired Student’s *t*-test to evaluate changes in hemoglobin (Hb) between baseline and the 3-month follow-up within each clinical setting and IHAT™ dosing regimen. *t* = Student’s *t* statistic; *p* = two-sided *p* value. AUB, abnormal uterine bleeding; Hb, hemoglobin. * Denotes statistically significant *p*-values (*p* < 0.05).

## Data Availability

The data presented in this study are available on reasonable request from the corresponding author. The data are not publicly available due to privacy and ethical restrictions.
